# Incidence and prevalence of functional neurological disorder: a systematic review

**DOI:** 10.1136/jnnp-2024-334767

**Published:** 2024-12-11

**Authors:** Sara A Finkelstein, Clare Diamond, Alan Carson, Jon Stone

**Affiliations:** 1 Functional Neurological Disorder Unit, Division of Behavioral Neurology and Integrated Brain Medicine, Department of Neurology, Massachusetts General Hospital, Boston, Massachusetts, USA; 2 Harvard Medical School, Boston, Massachusetts, USA; 3 Bristol Trials Centre, University of Bristol, Bristol, UK; 4 Centre for Clinical Brain Sciences, University of Edinburgh, Edinburgh, UK

## Abstract

**ABSTRACT:**

**Background:**

Robust epidemiological data regarding population incidence and prevalence of functional neurological disorder (FND) would be helpful with regards to resource allocation and planning for this disorder, particularly given high symptom burden and high healthcare utilisation. We therefore aimed to systematically review and synthesise available data on FND incidence and prevalence.

**Methods:**

PubMed was searched to identify original research articles that reported on the incidence or prevalence of FND. Risk of bias assessment for each study was conducted. Incidence and prevalence rates of FND were additionally estimated by extrapolating data from low risk of bias studies on functional seizures alone.

**Results:**

Thirty-nine articles were included. Nineteen reported on FND incidence, 21 reported on prevalence. Comparison between studies was difficult due to methodological differences and significant heterogeneity of incidence and prevalence estimates was found. The incidence of FND was estimated at 10–22/100 000, while minimum prevalence of FND was estimated at 80–140/100 000, with a possible range of 50–1600/100 000. Incidence of paediatric FND was estimated to be between 1 and 18/100 000.

**Conclusions:**

The range of incidence and prevalence varies widely across studies, with significant heterogeneity among studies and most studies likely provide underestimates due to methodological challenges. However, using our best method as a conservative estimate, there are likely a minimum of 50–100 000 people with FND in the UK, as an example country. Given that FND appears to be more prevalent than many other well-known and well-funded neurological disorders, incidence and prevalence data suggested here indicate the need for greater research and clinical funding allocation to FND programmes.

**WHAT IS ALREADY KNOWN ON THIS TOPIC**

- A number of studies have tried to delineate the incidence and prevalence of functional neurological disorder (FND).

**WHAT THIS STUDY ADDS**

- This study provides a contemporary synthesis of incidence and prevalence data on FND in a systematic review, with a best estimate of 50-100,000 people living with FND in the UK, as an example country.

**HOW THIS STUDY MIGHT AFFECT RESEARCH, PRACTICE OR POLICY**

- Having a more accurate estimate of FND incidence and prevalence would help direct appropriate healthcare policy, planning and resource allocation, as well as prioritisation of research funding across government agencies and foundations.

## Background

 Functional neurological disorder (FND) describes symptoms and signs of genuinely experienced alterations in the voluntary motor, somatosensory and perceptual systems, which are distressing or impairing, and manifest one or more patterns or deficits typical of the disorder. FND can manifest as a number of different symptoms, including (functional/non-epileptic) seizures, limb weakness, movement disorders (eg, tics, jerks, tremors, gait), speech, swallowing and communication disorders, dizziness (also known as persistent postural perceptual dizziness), somatosensory or special sensory changes and cognitive symptoms. Data show that individuals with FND comprise 5%–15% of patients presenting to neurology services.^1 2^ FND is often associated with a high degree of physical and psychological symptoms including comorbidity with chronic pain disorders, fatigue and anxiety/affective disorders.^3 4^

Epidemiological data, including both incidence and prevalence, can help determine how resources should be allocated in managing specific diseases and disorders. Incidence is defined as the number of new cases of a disease/disorder within a defined population, over a period of time. Prevalence is defined as the total number of cases of a disease/disorder within a population, and can be divided into period prevalence and lifetime prevalence. Period prevalence is the total number of active cases captured over a period of time, while lifetime prevalence is defined as the proportion of people within the population who experience the disease/disorder at any point in their lifetime.

A systematic review found mean annual costs related to FND to lie somewhere between US$5000 and US$87 000 per patient^5^ or >US$1.2 billion in annual emergency department and inpatient costs in the USA,^6^ suggesting that FND is associated with high healthcare utilisation. However, cost estimates and appropriate resource allocation for FND have been hampered by the fact that robust epidemiological data regarding population incidence and prevalence of this disorder are sparse, with no contemporary systematic reviews attempting to synthesise what data are available. Lack of clarity on the epidemiology of FND is likely due to a number of factors, including changes to the definition and method of diagnosis of FND over the past several decades, limited clinician knowledge and documentation bias, research that has generally focused on discrete symptom subtypes of FND (eg, functional seizures or functional motor disorders) and because it is a diagnosis that must usually be made in secondary care.

To address this gap in knowledge, we aimed to systematically review the available incidence and prevalence data on FND at a population level in order to make a determination regarding the minimum incidence and prevalence of this disorder, exploring sources of heterogeneity between estimates.

## Methods

### Eligibility criteria

To be included in this systematic review, studies had to: (i) be original research; (ii) report on patients with FND (described as functional or psychogenic neurological symptoms/disorder, hysteria or conversion disorder, or, in the case of functional seizures, dissociative or non-epileptic) in adults and/or children; (iii) report either on incidence or prevalence of FND, or provide data from which incidence and/or prevalence could be calculated, within a defined geographic area for which the population was known. Studies in which only one FND subtype (eg, functional seizures, functional movement disorder, functional limb weakness) were considered were included, but studies focused exclusively on functional cognitive disorder, persistent postural perceptual dizziness (PPPD), functional speech or functional sensory disturbances (loss of vision, hearing or tactile sensation) were not. PPPD and functional cognitive disorder were not included as these have only recently been defined as FND subtypes and in the case of the latter diagnostic guidelines have only recently been operationalised. Studies more broadly examining ‘functional’ or ‘somatoform’ disorders were included only if the data included specific frequency of FND symptoms such as functional motor symptoms or functional seizures. Search was not limited by date of publication or study quality. Studies looking at special subpopulations only (eg, refugees) and non-English language studies were excluded.

### Search strategy

A search of the PubMed database was updated to 15 August 2023. The reference lists of all relevant studies, as well as reference lists of relevant review articles identified in the PubMed search, were additionally screened (snowball search).

For the PubMed search, we defined two main search concepts of “functional neurological disorder” and “epidemiology”, and combined them. To search for articles related to FND, we searched for any of the terms “psychogenic”, “conversion disorder”, “non-epileptic”, “functional movement disorder” or “functional neurologic*” in the title/abstract. To search for articles related to epidemiology, we searched for any of the terms “incidence”, “prevalence” or “epidemiology” in the title/abstract.

### Study selection

A two-step selection process was completed by one reviewer (SAF) and used to identify studies for inclusion. First, all titles and abstracts were screened for relevance. Second, the full texts of the relevant studies were reviewed, and inclusion and exclusion criteria were applied. Reasons for exclusion were documented. In cases where it was unclear whether a study should be included, study was discussed among the author group with consensus reached. All studies meeting eligibility criteria, regardless of degree of risk of bias, were included in the synthesis of results.

### Data extraction

Data extraction was completed in duplicate by two independent reviewers (SAF and CD) using a standardised data collection form. Incidence and prevalence data were taken directly as reported from the studies and synthesised in data tables by study. In several cases, incidence and/or prevalence was not reported, but data on frequency of FND (or FND symptom subtype) and population of the catchment area were recorded, allowing incidence and/or prevalence to be calculated and reported in data tables by study. Bibliographic data (author, publication year), country, setting, patient age and sex, methods and source(s) of data ascertainment, inclusion and exclusion criteria and definitions/diagnostic criteria for FND (including for functional seizures whether electroencephalogram (EEG) confirmation was required) were additionally extracted. For the purposes of this review, any study including patients <16 years of age was considered to include a paediatric population. Information that was missing or unclear was marked as ‘not reported’.

### Risk of bias assessment

In accordance with the Preferred Reporting Items for Systematic Reviews and Meta-Analyses guidance for systematic reviews^7^ and because significant methodological heterogeneity was expected among studies, we used a component approach to assess study quality and risk of bias. Components that were deemed relevant to study quality were based on authors’ clinical and research knowledge of the field, as well as drawing from several checklists for assessing observational studies (Joanna Briggs Institute Critical Appraisal Checklist, Agency for Healthcare Research and Quality Methodology Checklist, Crombie’s items).^8^,^10^ Studies were evaluated in four domains: research methodology, cases captured, case definition and ascertainment and quality assurance (see table 1 for further description of domains). Each domain was rated using a qualitative traffic light system: green indicating low risk of bias, yellow indicating medium risk of bias and red indicating high risk of bias. Articles were systematically assessed in a standardised manner, and rechecked to ensure consistency of application of traffic light system across studies. Domains of ‘research methodology’, ‘cases captured’ and ‘case definition and ascertainment’ were given a ‘green light’ if all areas of interest within the domain were evaluated as high quality/low risk of bias OR if all areas but one were evaluated as high quality except for one rated as moderate quality/medium risk of bias. Domain was marked as a ‘yellow light’ if two or more areas of interest within the domain were evaluated as moderate quality/medium risk of bias. Domain was marked as a ‘red light’ if any of the areas of interest within the domain were evaluated as low quality/high risk of bias. For the fourth domain of ‘quality assurance’, a green light was given if there was any inclusion of a process designed to check quality of data once it had been collected, a yellow light was given if there was some part of the case selection process built in that would help assure good case selection and a red light was given if there was no mention of any method of quality check of the data or quality assurance built in to case selection process. If data were unknown or missing, a medium risk of bias was noted.

**Table 1:** Domains used to assess quality of studies

Domain	Considerations
A: Research methodology	Were selection criteria of patients clearly described?Were patients selected consecutively or randomly?Was the sample size adequate?Was data collected prospectively or retrospectively?Were statistics adequately explained and reproducible?
B: Cases captured	Was the geographical area well-defined?Were all relevant data sources included? (neurology, psychiatry, emergency care, etc)Was the response rate adequate (if applicable)?
C: Case definition and ascertainment	Was the case definition explicitly defined?Was the case definition for FND up-to-date?Was the case definition overly liberal or restrictive?Were cases ascertained by a neurologist/clinical assessment?Were incident cases truly incident?
D: Quality assurance	Was any assessment of quality assurance undertaken?

FND, functional neurological disorder.

### Data analysis

Assuming a normal distribution in the population sample, 95% CIs were calculated for prevalence studies where possible using the formula $p^{\prime }\pm Z\sqrt{\left(\frac{p^{\prime }(1-p^{\prime })}{n}\right)}$ where *p’* was the proportion of people with FND (number of people with FND divided by the total sample size, *n*).

### Data extrapolation

As we anticipated data heterogeneity and studies with high risk of bias, we additionally sought to estimate incidence and prevalence through other means. Studies with the highest methodological quality, based on risk of bias assessment, reporting on incidence or prevalence of functional seizures alone, were used to extrapolate overall FND incidence and prevalence (ie, encompassing seizure, motor, sensory subtypes, although not necessarily accounting for more newly defined FND subtypes including functional cognitive disorder and persistent postural perceptual dizziness). This was accomplished by assuming that functional seizures represent 30% of all patients with FND, determined by a weighted average (based on number of patients in each study taken) derived from 13 studies with relatively larger samples sizes where different FND subtypes including functional seizures were documented.^11^,^24^ Of note, there may be differences in percentage of functional seizures between paediatric and adult populations, geographic/ethnocultural groups or depending on when the study was conducted; however, this seemed a reasonable estimate based on looking at a number of studies with heterogeneous populations, and correlated well with the authors’ own clinical observations.

## Results

**Figure 1:**
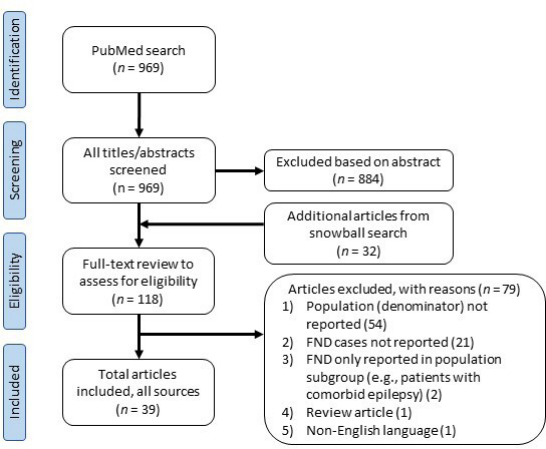
Study selection flow chart. FND, functional neurological disorder.

The PubMed search returned 969 unique abstracts published between 1956 and 15 August 2023. Based on a review of the abstracts, 884 articles were excluded because they did not relate to the research question, were review articles or were non-English language articles. The full texts of the remaining 85 articles were reviewed, of which 24 were determined to meet inclusion criteria. Reasons for exclusion were documented (figure 1). A snowball search was conducted by reviewing the reference lists of included articles as well as reference lists of relevant review articles found during the initial search. This yielded a further 32 articles that underwent full-text review; 17 were excluded with reasons for exclusion documented, while the remaining 15 met inclusion criteria. This yielded a combined total of 39 included articles (see figure 1 for summary of study screening and identification).

### Incidence

A total of 19 studies reported on incidence of FND; seven studies did not limit results by FND symptom type,^20^,^26^ nine studies reported only on functional seizures,^27^,^35^ two reported only on functional limb weakness^36 37^ and one study reported only on stroke-like symptoms.^38^ Of the studies not limiting results by symptom subtype, two documented in detail FND symptoms that were present (including seizures, dizziness, movement disorders, weakness, somatosensory, speech, visual and auditory symptoms),^22 24^ three documented presenting/main FND symptom as motor (including movement disorders and weakness), seizure, sensory or mixed^20 21 23^ and one did not characterise patient symptoms.^26^ Studies from Europe, North America and Australia/New Zealand were represented and included both adult and paediatric populations. Incidence rates and demographic data from these studies can be found in table 2. For studies that reported incidence rates for each year of the study separately in addition to the overall average incidence rate by year, only the average incidence rate by year was reported in the table.

**Table 2:** Summary of studies of incidence of FND

Study	Country	Cases	Population	Mean age at diagnosis (SD); age range	% F	Incidence/100 000(95% CI)*
**All FND†—adult and paediatric‡**						
Stefánsson *et al*^26^	USAIceland	1374200	710 917§204 578§	NR; <14 to >65NR; <14 to >65	6883	22.011.0
**All FND—adult only**						
Beharry *et al*^20^	New Zealand	125	369 000	40 (17); NR	69	11
Stevens^25^	UK	164	512 200	NR; NR	NR	10.7¶
**All FND—paediatric only (<16 years**)						
Yong *et al*^24^	UK	64	116 720	13; 5–15	70	18.3 (13.8 to 22.8)
Raper *et al*^21^	UK	124	600 000	NR; 4–19	56	6.0
Ani *et al*^22^	UK	204	12 542 420	12.5; 7–15	76	1.30 (1.11 to 1.52)
Kozlowska *et al*^23^	Australia	194	4 250 000	11.8 (2.7); 3.1–15.9	71	2.3 (2.0 to 2.6) (Aus)4.2 (3.5 to 5.0) (NSW)
**Motor FND—adult only**						
Stone *et al*^36^	UK	107	800 000	39.1; NR	79	3.9
Binzer *et al*^37^	Sweden	30	260 000	38.8; NR	60	4.8¶
**Functional seizures—adult and paediatric**						
Villagrán *et al*^27^	Norway	79	265 238	27; 11–78	77	3.1 (2.4 to 3.7)
Duncan *et al* (age 13+ years)^28^	UK	68	367 566	32.6; 14.0–71.1(at onset)	82	4.90
O’Sullivan *et al*^29^	Ireland	50	1 100 000	32.4 (11.8) FS34.8 (11.1) FS and epilepsy	61	0.91
Kotsopoulos *et al* (age 14+ years)^30^	The Netherlands	63	190 860	NR; NR	67	19.8¶
Sigurdardottir and Olafsson (age 15+ years)^31^	Iceland	14	1 000 955	27.6; 16–54	79	1.4
**Functional seizures—adult only**						
Lehn *et al*^35^	Australia	46114	1 600 000	33; 23.25–44.534.5; 26–49	4163	11.5¶ (‘all comers’)7.1¶ (‘status’)
Szaflarski *et al*^32^	USA	77	600 000	37.2; 16 to >65	73	3.03
Forsgren^33^	Sweden	4	191 356	NR; NR	NR	2.4¶
**Functional seizures—paediatric only (<18 years**)						
Hansen *et al*^34^	Denmark	386	NR**	15.7; NR	83	2.4
**Stroke-like symptoms—adult only**						
Gargalas *et al*^38^	UK	98	900 000	49.1 (18.8); NR	63	10.9¶

* Where reported.

† ‘All FND’ indicates studies that did not limit results by FND symptom type.

‡ Unless otherwise specified in table, studies including pediatricpaediatric populations did not specify a minimum age cutoffcut-off.

§ Population as reported at the end of study, which spanned over 10 years. Lower population at beginning of study period was taken into account when calculating overall incidence.

¶ Incidence calculated based on data provided in study.

** Population not reported but was stated as equal to the total population of persons aged 5–17 years in Denmark, based on the Civil Person Registration number, which is provided to every person born or immigrating to Denmark.

Aus, Australia; CI, Confidence interval; F, female; FND, functional neurological disorder; FS, functional seizures; NR, not reported; NSW, New South Wales; SD, Standard deviation.

Of the three studies that did not limit results by FND symptom type (including both motor symptoms and seizures, among other symptoms in some cases) in either adult or combined adult and paediatric populations, similar incidence was reported across studies (10.7–22/100 000).^20 25 26^ All three used only one data source, although data sources varied (psychiatric case registry^26^ vs single neurologist^25^ vs patients presenting to hospital).^20^ Interestingly, within a single study by Stefansson *et al*, using similar data sources (psychiatric registries)—one from Iceland and one from the USA—the incidence rate of one population was double the other.^26^ In the case of the study by Stevens, while only data from a single neurological practice was used, the author notes that they were in the unique position of being the only neurologist in the district, and as such presumably all referrals would have been directed to them. Case definitions varied significantly across studies ranging from ‘hysterical neurosis’^26^ to ‘neurological symptoms without recognised disease’,^25^ to use of an in-house coding system for functional neurological symptoms also inclusive of hypochondriasis.^20^ Of interest, incidence of multiple FND subtypes combined, across these three studies, overlaps with incidence from several studies looking at incidence of functional seizures alone.

Of the four studies reporting on incidence of that did not limit results by FND symptom type in paediatric populations alone, incidence ranged from 1.3 to 18.3/100 000.^21^,^24^ While all four studies included multiple data sources for cases, the study with the highest incidence rate (Yong *et al*) was the only one to include paediatric neurology as a data source; notably, 77/97 cases (79%) were derived from paediatric neurology sources in this study (other cases from general paediatricians and psychologists).^24^ The study by Yong *et al* was also the only one conducted in the wake of the COVID-19 pandemic, while the other studies were conducted in the 15 years prior.

Of the nine studies reporting on incidence of functional seizures alone, in either adult only or a combined adult and paediatric population, incidence ranged from 0.91 to 19.8/100 000.^27^,^35^ Studies reporting the highest incidence of functional seizures used either multiple data sources^30^ or data from the emergency department,^35^ while studies reporting the lowest incidence used only data from video-EEG/telemetry units (including long-term monitoring epilepsy unit).^29 31^ Notably, in the study by Sigurdardottir *et al*, which reported the second-lowest incidence, only cases in which patients were having events that ‘resembled epileptic seizures’, where there was diagnostic uncertainty, were considered. Studies with the highest reported incidence also considered both clinical and EEG data in order to determine diagnosis of functional seizures,^28 30 35^ while studies with lowest reported incidence only considered cases of EEG-documented functional seizures.^29 31^

### Prevalence

Twenty-one studies reported on prevalence rates, 16 on period prevalence^2739^,^53^ and six on lifetime prevalence.^53^,^58^ Of the period prevalence studies, nine studies did not limit results by FND symptom type,^39^,^4552 53^ four reported on functional seizures only,^27 47 48 51^ one reported on functional seizures and functional weakness,^46^ one reported on functional parkinsonism only^49^ and one reported on functional dystonia only.^50^ Of the studies not limiting results by symptom subtype, one documented in detail FND symptoms that were present (including seizures, weakness, movement disorders, somatosensory and speech symptoms),^40^ two documented primary FND symptom (seizure or motor)^45 52^ and six did not characterise patient symptoms.^3941^,^44 53^ Of the lifetime prevalence studies, five did not limit results by FND symptom type^53^,^57^ and one reported on functional seizures only.^58^ Of the studies not limiting results by symptom subtype, two documented in detail FND symptoms that were present (including syncope/seizure, movement disorders, weakness, dizziness, swallowing, speech, visual, somatosensory and auditory symptoms)^55 57^ and three did not characterise patient symptoms.^53 54 56^ Studies reporting on data from Europe, the Middle East, Africa, South America and Asia were represented, as were both adult and paediatric populations. Prevalence rates and demographic data from these studies can be found in tables3  4.

**Table 3:** Summary of studies of period prevalence of FND

Study	Country	Study period	Cases	Population	Mean age at diagnosis (SD); age range	% F	Prevalence/100 000(95% CI)*
**All FND†—adult and paediatric‡**							
Jha *et al*(age 13+ years)^42^	Nepal	1 month	20	1647	NR; NR	NR	1214§ (685.4 to 1743.3)*
Singh and Lee^40^	UK	NR	18	37 000	38; 26–74	78	48 (22.0 to 64.4)*
Faravelli *et al*(age 14+ years)^41^	Italy	6 months	2	673	NR; NR	100	297§ (−114.1 to 708.4)*
Elnagar *et al*^52^	India	3 months	2	1383	NR; NR	50	144.5 (−55.8 to 344.8)*
Dube^53^	India	1.5 years	179	600 000	NR; NR	NR	607 (518.3 to 695.7)*
Helgason^39^	Iceland	NR	22	5395	NR; NR	73	408§ (237.7 to 577.8)*
**All FND—ault only**							
Fink *et al*^43^	Denmark	NR	3	191	NR; NR	67	1571§ (−192.6 to 3334.6)*
Nandi *et al*^44^	India	NR	28	4053	NR; NR	93	691‡ (435.8 to 945.8)*
**All FND—paediatric only**							
Chandra *et al*(age 5–13 years)^45^	India	1.5 years	31	65 244	NR; NR	42	48§ (30.8 to 64.2)*
**Functional limb weakness—adult and paediatric**							
Rief *et al*(age 14+ years)^46^	Germany	NR	41	2050	NR; NR	NR	2000§ (1394.0 to 2606.0)*
**Functional seizures—adult and paediatric**							
Ferruzzi *et al*^51^	Brazil	5 years	30	2994	NR	NR	1002§ (645.2 to 1647.8)*
Villagrán *et al*^27^	Norway	10 years	63	265 238	27; NR	76	23.8 (17.9 to 29.6)
Inaida *et al*^47^	Japan	10 years	413	1 600 000	M: 37.0, F: 34.5; 1–74	53	25.8§ (23.3 to 28.3)*
Rief *et al*(age 14+ years)^46^	Germany	NR	41	2050	NR; NR	NR	2000§ (1394.0 to 2606.0)*
**Functional seizures—paediatric only**							
Mohamed *et al* (age 6–14 years)^48^	Sudan	6 months	15	74 949	NR; NR	NR	20§ (9.9 to 30.1)*
**Functional parkinsonism—adult only**							
Polara *et al*^49^	Switzerland	10 years	12	470 512	45.5; NR	67	0.64 (−0.1 to 1.4)*
**Functional dystonia—adult only**							
Dressler *et al*^50^	Germany	N/A¶	15	525 731	47.2 (20.8)	48	2.8 (1.4 to 4.2)*

* Confidence intervalCI calculated (not provided in study).

† ‘All FND’ indicates studies that did not limit results by FND symptom type.

‡ Unless otherwise specified in table, studies including pediatricpaediatric populations did not specify a minimum age cutoffcut-off.

§ Prevalence calculated based on data provided in study.

¶ Point prevalence study – —looked at all cases active on date of database inquiry.

CI, Confidence interval; F, female; FND, functional neurological disorder; M, male; N/A, not applicable; NR, not reported; SD, Standard deviation.

**Table 4:** Summary of studies of lifetime prevalence of FND

Study	Country	Cases	Population	Mean age at diagnosis (SD); age range	% F	Prevalence/100 000(95% CI)*
**All FND†—adult and paediatric‡**						
Choi *et al*^56^	South Korea	~20 (NR)~30 (NR)	65106027	NRNR	NRNR	300 (KECA-R) (167.2 to 432.8)*500 (KECA-2011) (321.9 to 678.1)*
Deveci *et al*^55^	Turkey	61	1086	NR; 15–65	87	5617 (4247.6 to 6986.4)*
Lieb *et al*(age 14–24 years)^54^	Germany	8	2548	NR; 14–24	88	314 (96.8 to 531.2)*
Dube^53^	India	261	29 468	24.4 (8.3); 16.1–32.7	96	886 (779 to 993)*
**All FND—paediatric only**						
Essau *et al*(age 12–17 years)^57^	Germany	14	1035	NR; 12–17	71	1353 (649.2 to 2056.8)*
**Functional seizures—adult and paediatric**						
Farghaly *et al*^58^	Egypt	14	62 583	NR; NR	NR	22 (10.3 to 33.7)*

* Confidence intervalCI calculated (not provided in article).

† ‘All FND’ indicates studies that did not limit results by FND symptom type.

‡ Unless otherwise specified in table, studies including pediatricpaediatric populations did not specify a minimum age cutoffcut-off.

CI, Confidence interval; FND, functional neurological disorder; KECA-2011, Korean Epidemiological Catchment Area study 2011; KECA-R, Korean Epidemiological Catchment Area study Replication; NR, not reported; SD, Standard deviation.

Of the eight studies reporting on prevalence that did not limit results by FND symptom type, in either adult only or in a combined adult and paediatric population, prevalence ranged from 48 to 1571/100 000. Notably, the study reporting the lowest prevalence (48/100 000) by Singh and Lee,^40^ reported that their method of case finding, which entailed asking general practitioners to recall patients they had seen with FND (without chart review) was likely to be unreliable. Two other studies^41 52^ with lower prevalence (144.5 and 297/100 000, respectively) only had two FND cases each. The study with the highest prevalence (1571/100 000) by Fink *et al*, used prospective data collection from general practices and encompassed both a patient survey and interview by a psychiatrist.^43^ Studies with the next three highest prevalence rates (607–1214/100 000)^42 44 53^ gathered data through door-to-door community surveys asking about symptoms, with two^44 53^ involving an interview/examination by a psychiatrist to confirm cases but no involvement from neurology.

Of the four studies reporting on prevalence of functional seizures separately, in combined adult and paediatric populations, prevalence ranged from 23.8 to 2000/100 000. In one study with a lower prevalence (25.8/100 000) by Inaida *et al*, only cases in which an epilepsy diagnosis was revised to a functional seizure diagnosis were considered.^47^ The study with the lowest prevalence (23.8/100 000), by Villagrán *et al*, used a patient registry and included cases with the International Classification of Disease, 10th edition diagnostic code for either functional seizures (F44.5) or ‘convulsions not elsewhere classified’ (R56.8). The studies with higher prevalence used either patient self-report obtained through community survey alone (Rief *et al*—2000/100 000)^46^ or a community survey that also incorporated clinical and EEG data (Ferruzzi *et al*—1002/100 000).^51^ Notably, the study by Rief *et al* also reported the prevalence of functional limb weakness to be 2000/100 000, which, if taken together with the functional seizure prevalence, would indicate a minimum prevalence of 4000/100 000 for FND more broadly. None of the studies required EEG-documented functional seizures for case inclusion.

### Risk of bias assessment

Risk of bias for both incidence and prevalence studies across four domains is summarised in tables5  6.

**Table 5:** Risk of bias assessment of functional neurological disorder incidence studies

Study	Data collection	Case source	Case definition	Case ascertainment	Risk of bias			
					Research methods	Cases captured	Case definition and ascertainment	Quality assurance
Ani *et al*^22^	Prospective	Paediatrics, psychiatry	DSM-IV	Clinician questionnaire	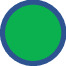	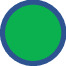	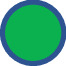	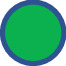
Beharry *et al*^20^	Retrospective	Neurology	In-house coding system	Chart review	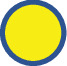	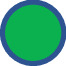	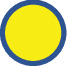	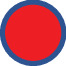
Binzer *et al*^37^	Prospective	Neurology	DSM-III/IV	Clinical* (neuro)	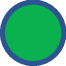	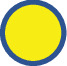	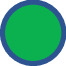	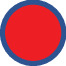
Duncan *et al*^28^	Prospective	Neurology	EEG† or clinical	Clinical* (neuro)	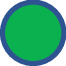	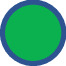	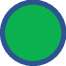	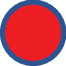
Forsgren^33^	Prospective	Neurology, primary care, internal medicine	NR	Chart review±clinical (neuro)	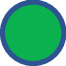	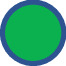	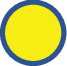	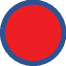
Gargalas *et al*^38^	Retrospective	Emergency department	NR	Chart review, clinical* (neuro)	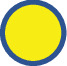	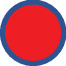	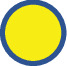	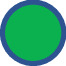
Hansen *et al*^34^	Retrospective	Paediatrics	ICD-10 F44.5, DSM-5	Database	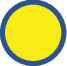	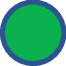	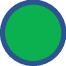	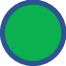
Kotsopoulos *et al*^30^	Prospective	Primary care, neurology	NR	Chart review±clinical* (neuro)	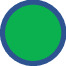	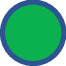	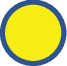	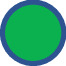
Kozlowska *et al*^23^	Prospective	Paediatrics, psychiatry	c/w DSM-5	Case registry	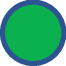	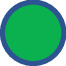	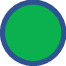	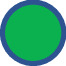
Lehn *et al*^35^	Retrospective	Emergency department	Clinical	Database, chart review	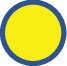	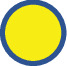	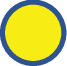	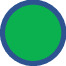
O’Sullivan *et al*^29^	Retrospective	Neurology	EEG†	vEEG record	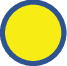	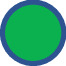	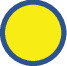	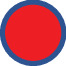
Raper *et al*^21^	Retrospective	Neurology	ICD-10 F44; 45; 48	Database	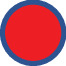	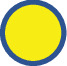	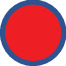	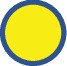
Sigurdardottir and Olafsson^31^	Retrospective	Neurology	EEG†	vEEG record	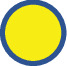	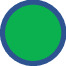	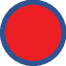	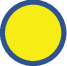
Stefánsson *et al*^26^	Retrospective	Psychiatry	DSM-II	Database, chart review	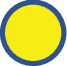	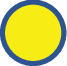	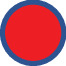	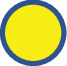
Stevens^25^	Prospective	Neurology	NR	Database	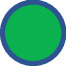	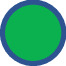	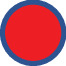	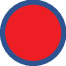
Stone *et al*^36^	Prospective	Neurology	c/w DSM-5	Clinical* (neuro)	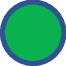	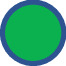	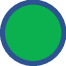	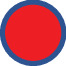
Szaflarski *et al*^32^	Retrospective	Neurology	EEG†	Chart review	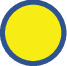	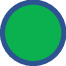	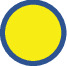	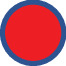
Villagrán *et al*^27^	Retrospective	All health services	EEG† or clinical	Chart review	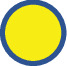	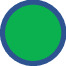	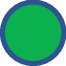	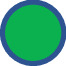
Yong *et al*^24^	Prospective	Paediatrics, neurology, psychiatry	DSM-5	Clinical* (peds neuro, peds), chart review	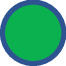	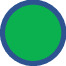	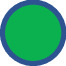	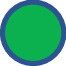

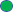

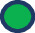
=Low risk of bias; 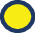 = =medium risk of bias; 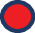 = =high risk of bias – —please see ‘Mmethods’ section for further details on how this was operationalizsed.

* Clinical examination performed, with data in parentheses indicating area of expertise of person(s) performing examination (neuro – neurology, peds neuro – pediatric neurology, peds – pediatrics).

† EEG-documented.

c/w, consistent with; DSM-II/III/IV/5, Diagnostic and Statistical Manual of Mental Disorders, second through fifth editions; ICD-10, International Classification of Disease, 10th edition; neuro, neurology; NR, not reported; peds, paediatrics; peds neuro, paediatric neurology; (v)EEG, (video)electroencephalogram.

**Table 6:** Risk of bias assessment of functional neurological disorder prevalence studies

Study	Data collection	Case source	Case definition	Case ascertainment	Risk of bias			
					Research methods	Cases captured	Case definitionand ascertainment	Quality assurance
Chandra *et al*^45^	Prospective	Primary care	DSM-III-R	Symptom checklist, evaluation by paediatrics and mental health	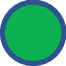	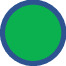	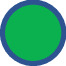	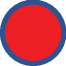
Choi *et al*^56^	Prospective	Door-to-door survey	DSM-IV	Structured interview	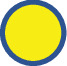	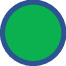	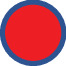	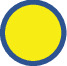
Deveci *et al*^55^	Prospective	Door-to-door survey	DSM-IV-TR	Structured interview, clinical* (neuro)	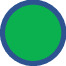	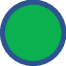	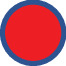	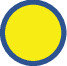
Dressler *et al*^50^	Retrospective	Database, neurology	c/w DSM-5	Chart review	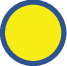	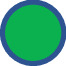	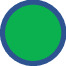	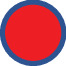
Dube^53^	Prospective	Door-to-door survey	Hysterical neurosis	Structured interview, clinical (psych)	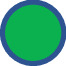	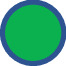	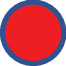	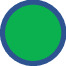
Elnagar *et al*^52^	Prospective	Door-to-door survey	Hysterical neurosis	Structured interview, clinical* (psych)	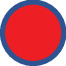	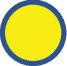	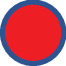	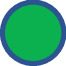
Essau *et al*^57^	Prospective	Door-to-door survey	DSM-IV	Structured interview, parent and patient survey	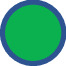	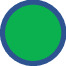	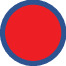	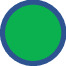
Faravelli *et al*^41^	Prospective	Door-to-door survey	DSM-III	Structured interview	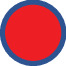	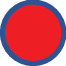	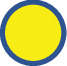	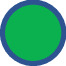
Farghaly *et al*^58^	Prospective	Door-to-door survey	EEG†	Structured interview, EEG, clinical* (neuro)	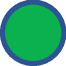	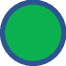	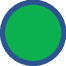	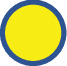
Ferruzzi *et al*^51^	Prospective	Primary care	NR	Patient survey, clinical history, collateral history, EEG	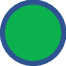	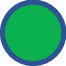	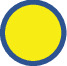	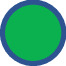
Fink *et al*^43^	Prospective	Primary care	DSM-IV	Patient survey, structured interview	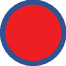	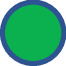	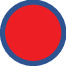	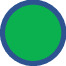
Helgason^39^	Retrospective	Primary care	Hysterical neurosis	PCP interview, patient survey, collateral history	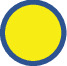	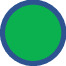	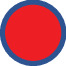	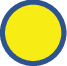
Inaida *et al*^47^	Retrospective	Database, all care levels	ICD-10 F44 code	vEEG review	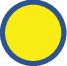	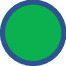	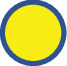	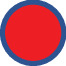
Jha *et al*^42^	Prospective	Door-to-door survey	NR	Structured interview	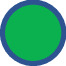	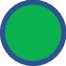	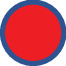	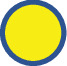
Lieb *et al*^54^	Prospective	Door-to-door survey	DSM-IV	Patient survey	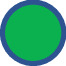	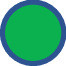	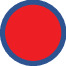	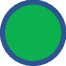
Mohamed *et al*^48^	Prospective	Door-to-door survey	NR	Identification by teachers, clinical* (neuro)	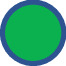	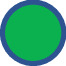	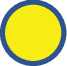	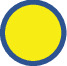
Nandi *et al*^44^	Prospective	Door-to-door survey	Hysterical neurosis	Interview by psychiatrist	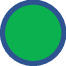	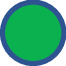	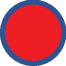	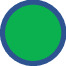
Frasca Polara *et al*^49^	Retrospective	Neurology	Fahn and Williams FMD criteria	Chart review	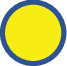	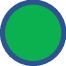	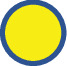	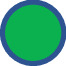
Rief *et al*^46^	Prospective	Door-to-door survey	Symptom-based	Patient survey	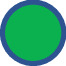	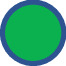	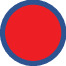	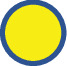
Singh and Lee^40^	Prospective	Primary care	DSM-III-R‡	Chart review	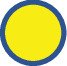	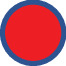	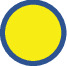	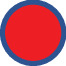
Villagrán *et al*^27^	Retrospective	Database, all care levels	EEG† and clinical	Chart review	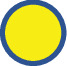	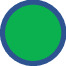	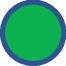	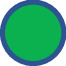

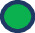
=Low risk of bias; 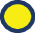 = =medium risk of bias; 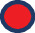 = =high risk of bias – —please see ‘Mmethods’ section for further details on how this was operationalizsed.

* Clinical examination performed, with data in parentheses indicating area of expertise of person(s) performing examination (neuro – neurology, psych - psychiatry).

† EEG-documented.

‡ Modified DSM-III-R case definition that excluded preceding stressor.

c/w, consistent with; DSM-III/III-R/IV/IV-TR/5, Diagnostic and Statistical Manual of Mental Disorders, 3rd edition, 3rd edition-revised, 4th edition, 4th edition-text revision, 5th edition; FMD, functional movement disorder; ICD-10, International Classification of Diseases, 10th edition; NR, not reported; PCP, primary care provider; vEEG, (video)electroencephalogram.

Multiple case definitions for FND were used across studies, including older Diagnostic and Statistical Manual of Mental Disorders (DSM) definitions and the entity of ‘hysterical neurosis’. For incidence studies, of the 11 studies not assessing functional seizures alone, three used a case definition consistent with the current DSM-5 diagnostic criteria.^23 24 36^ For prevalence studies (excluding those assessing functional seizures alone), one used a definition consistent with current DSM-5 criteria for FND.^50^ In general, the more up-to-date the definition used for the study, the lower the risk of bias with regard to case ascertainment was deemed to be, as it was likely both that case definitions overlapped with other conditions as they are currently defined and that cases were missed due to the strong focus on psychiatric morbidity needing to be present. Of the studies considering only functional seizures, three of nine incidence studies^27 28 35^ and two of five prevalence studies^27 47^ considered cases that were either EEG-documented or based on clinical diagnosis (of note, whether EEG-documented diagnosis only was considered was unclear in two of the other prevalence studies). Functional seizure studies that only considered EEG-documented cases were paradoxically considered to have higher risk of bias in that they likely underestimated incidence because accessing EEG confirmation is often not possible—either due to infrequent events or lack of access to services—and those accessing EEG services are often patients in which diagnosis of epilepsy versus functional seizures is more uncertain (potentially excluding more clear-cut cases of functional seizures). As noted in the results, studies with lower incidence of functional seizures all required EEG-documented events, while studies that considered both EEG and clinical data for case inclusion reported higher incidence. Validity of inclusion of patients without EEG-documented functional seizures is demonstrated by other work in the field, most notably, a large randomised controlled trial of treatment for functional seizures (Cognitive Behavioural Therapy for Adults with Dissociative Seizures or CODES trial) that included this group (for further discussion of issues surrounding this, please see referenced study).^59^

Case ascertainment methods, which speak to accuracy of diagnosis, included review of database diagnostic codes, chart review, patient self-report, structured interviews and clinical examination. Studies in which patient self-report was the primary method of case ascertainment were considered to have a high risk of bias, as they likely overestimated prevalence of FND. Six of the 10 prospective incidence studies^24 28 30 36 37 60^ and three of the 16 prospective prevalence studies^48 55 58^ employed clinical examination by a neurologist in order to confirm the diagnosis. Studies in which risk of bias was considered to be lowest for case ascertainment employed a multistep approach in which potential cases were identified through surveys or other methods, followed by a clinical examination to confirm the diagnosis.^45 48 55 58^

Sources of cases, which speak to opportunity to capture all potentially relevant cases within the sample but not accuracy of diagnosis, varied; for incidence studies, the majority used either neurology or psychiatry secondary care populations. Three incidence studies derived cases from emergency department populations,^20 35 38^ while five incidence studies included primary care sources (including paediatrics).^22^,^2430 34^ Cases derived from multiple sources were considered to be the most likely to capture all relevant patients. Cases derived from neurology populations were also considered likely to capture the majority of relevant patients given patients with FND primarily present with neurological symptoms, while acknowledging that these studies were still likely to miss a proportion of cases being seen only by psychiatry, primary care, etc. Nearly all of the prevalence studies used prospective data collection, with the most common method of data collection being a door-to-door community-based survey. While this had the advantage of screening a large number of people (and in some cases essentially screening everyone) in the population of interest for FND, many of these studies did not use rule-in physical examination signs to confirm the diagnosis nor involve consultation with a neurologist or neuropsychiatrist (with some exceptions).^48 49 51 55 58^ Other sources of data for prevalence studies included primary care, healthcare databases and neurology.

Precision of point estimates with regard to prevalence studies was difficult to ascertain (tables3  4). The majority of studies did not report CIs for prevalence estimates and instead estimates were calculated by the authors of this review. Of note, calculations were based on an assumed normal distribution, which could not be confirmed as data sets and SD were also not provided for included studies.

### Estimated FND incidence and prevalence based on rate of functional seizures

Of incidence studies looking at rates of functional seizures alone, studies by Duncan *et al*^28^ and Villagrán *et al*^27^ were determined to have the lowest risk of bias in populations including both children and adults, while the study by Hansen *et al*^34^ had the lowest risk of bias in the paediatric population. Assuming that patients with functional seizures represent approximately 30% of all patients with FND (see rationale and references in ‘Methods’ section), extrapolating from the functional seizure incidence rates from the aforementioned studies yielded an overall estimated incidence of FND between 10 and 16/100 000 for all ages, and 8/100 000 for the paediatric population.

Of studies looking at prevalence rates of functional seizures alone in both adults and children, the study by Villagrán *et al* was determined to have the lowest risk of bias. Extrapolating from the functional seizure prevalence rate from this study, assuming that patients with functional seizures represent approximately 30% of all patients with FND, yielded an estimated prevalence of FND of 79/100 000.

### Evidence synthesis

Due to high heterogeneity, a meta-analysis was not possible. The outcomes of the review were a review of the range of findings of incidence and prevalence, as well as data extrapolation.

## Discussion

**Figure 2:**
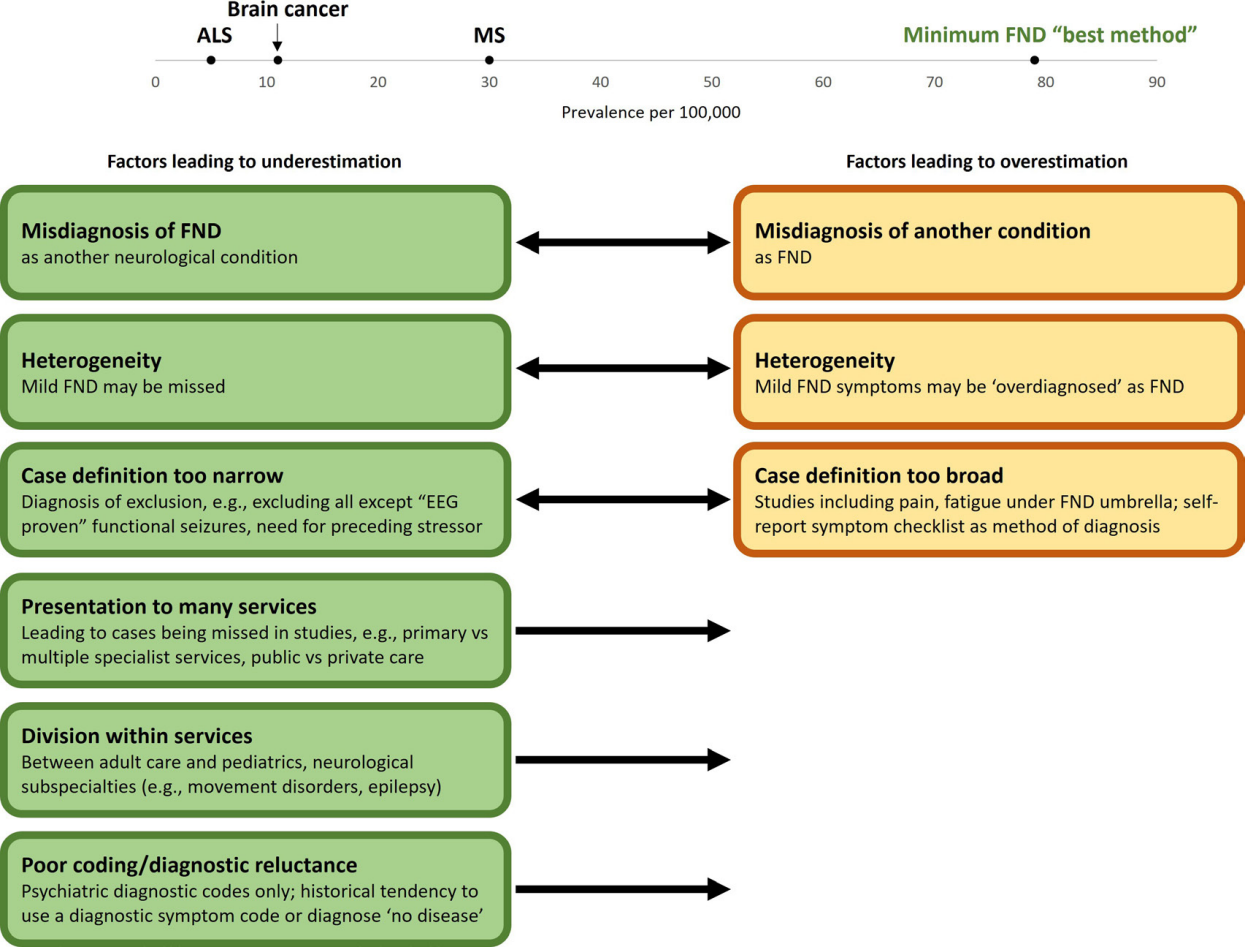
Factors leading to underestimation or overestimation of prevalence of functional neurological disorder (FND). ALS, amyotrophic lateral sclerosis; EEG, electroencephalogram; MS, multiple sclerosis.

Many barriers exist to assessing incidence and prevalence and then accurately aggregating data from FND studies (figure 2). The diagnostic criteria for FND have changed over time, and significant changes have occurred between the DSM-IV-text revision (and older versions of the DSM) and DSM-5 that likely led to a change in how many cases were being captured: shift from a ‘rule out’ to ‘rule in’ diagnosis with positive signs on examination and dropping the need for a stressor preceding symptom onset. As such, diagnostic precision as to what would be considered FND based on current diagnostic criteria likely was increasingly poor the older the study, leading to underestimation of cases in some instances (eg, due to only including cases with a preceding stressor, which, as an example, in one study led to exclusion of about one-third of cases)^55^ and overestimation in others (eg, including non-core FND symptoms such as pain under the umbrella of conversion disorder). In many of the included studies, neurologists or neuropsychiatrists were not involved in diagnosis, likely lowering precision. Diagnostic coding, which in many studies was used to identify cases, is likely inconsistent in clinical practice, with some patients possibly being lumped under the broader category of ‘somatoform disorders’, and some patients perhaps not having a diagnostic code for FND applied (eg, coding functional seizures less specifically as ‘convulsions’ or functional leg weakness as ‘leg weakness no cause’). Other reasons for diagnostic coding inconsistency may include lack of clinician knowledge regarding the diagnosis or confidence in making the diagnosis, or reluctance to apply what some view as a stigmatising label to a chart record. FND has also been shown to be underdiagnosed—for example, 8% of patients diagnosed and treated for status epilepticus in two large epilepsy trials were ultimately discharged with a diagnosis of prolonged functional seizures.^61^ There is also the more general barrier to assessing incidence and prevalence accurately due to logistical difficulties in obtaining large sample sizes. While several studies in this review had drawn from populations in the 100 000s or even in some cases >1 million range, many others were much smaller. By comparison, if we examine epidemiological studies of other neurological and psychiatric disease, overall population may be much larger—such as in a study examining prevalence of multiple sclerosis that included data from over 100 million people^62^—or on par with some of the studies included in this review—such as in a study examining prevalence of depression, in which around 400 000 participants were surveyed.^63^

More recently, as functional neurological symptoms have become increasingly recognised, there has been a trend towards compartmentalising them by neurological subspecialty, and roughly half of the studies included in this review reported data on patients with only one type of FND symptom (eg, functional seizures). This is out of keeping with how many patients with FND present, that is, with a mixed symptom picture, and makes estimates of FND symptom subtypes, such as functional seizures, more difficult to ascertain. Case ascertainment was also often from only one point of healthcare access that patients with FND are likely to encounter (eg, only considering cases from psychiatry), which will likely lead to epidemiological underestimation as patients with FND present to a wide array of providers including primary care and many specialists such as emergency physicians, internists, stroke specialists, orthopaedic surgeons, physiatrists, neurologists or psychiatrists. Furthermore, even if only examining cases from, for example, neurology, capturing all cases presenting to a given specialty within a defined geographic area presents its own challenges (eg, in the case of parallel public and private healthcare systems), with a number of studies likely missing a large number of cases. There was significant variability with respect to age range included, and some studies used a population denominator of the entire population for incidence and prevalence calculations, while the numerator only considered adult cases. There were studies included from a wide geographic, socioeconomic and cultural range, which may have introduced additional heterogeneity. Socioeconomic and cultural factors such as stigmatisation of FND on both the part of patients and healthcare providers (that may have affected willingness to present to healthcare and/or code for the disorder), the lens through which FND symptoms are understood, access to care and threshold for seeking out care, may have all influenced epidemiological estimates. These sources of heterogeneity make comparison of data between studies challenging. Looking at all the factors above we consider that the existing data are likely to lead to an underestimate of true incidence and prevalence rates.

### Incidence of FND

There was only one study looking at incidence in all age groups that did not limit results by FND symptom type,^26^ which found disparate rates between Icelandic and American populations, of 11 and 22 per 100 000, respectively. This study was conducted in 1976, using an out-of-date definition for FND and taking data only from psychiatric registries. Two additional studies looking at incidence of FND without limiting by symptom type in adults both determined similar incidence rates of 11/100 000.^20 25^ However, the risk of bias in case ascertainment was high in the study by Stevens^25^, and the study by Beharry *et al*^20^ likely included a high number of cases that were not true incident cases (ie, not the first presentation of FND). When data were extrapolated from functional seizure studies with a lower risk of bias, incidence for FND appeared to be somewhat similar, between 10 and 16/100 000. Synthesising these data, a reasonable estimate for *minimum* incidence of FND is 10–22/100 000. A reasonable estimate for the minimum incidence of functional seizures alone, based on lowest risk of bias studies, is 3–5/100 000.

Looking at paediatric data, low risk of bias studies that did not limit results by FND symptom type by Ani *et al*^22^ and Kozlowska *et al*^23^ reported incidence between 1 and 4/100 000. Incidence was notably higher in a more recent study by Yong *et al*, also noted to have low risk of bias, in which the rate of paediatric FND was found to be 18/100 000 with a sample taken from a secondary care paediatric neurology service only. The higher rate may represent decreased barriers to diagnosis over time, such as through increased awareness of FND among healthcare providers. This study was also conducted during the COVID-19 pandemic, and it has previously been reported that incidence of FND may have increased during this period.^64^ When data were extrapolated from Hansen *et al*,^34^ a study on functional seizures alone in the paediatric population with the lowest risk of bias, incidence was estimated at 8/100 000. Taking these data together, incidence of FND in the paediatric population alone was between 1 and 18/100 000, and likely at the higher end of that estimate.

### Prevalence of FND

Prevalence between studies was difficult to compare, as many studies had high risk of bias with respect to case definition, ascertainment and completeness or appropriateness of cases captured (either due to likely overestimation or underestimation of cases). Period prevalence rates varied by two orders of magnitude, ranging from 10s to 1000s per hundred thousand. Calculating the point prevalence by extrapolating from a ‘low risk of bias’ study looking at prevalence of functional seizures alone yielded an estimate of 79/100 000, which may provide a more accurate estimate. Looking at the studies themselves, if we exclude the study with the lowest reported prevalence (48/100 000) by Singh and Lee^40^ (which is likely reasonable given the presumed gross underestimate of cases as reported by the authors), the next lowest estimate is 144.5/100 000 (Faravelli *et al*). Synthesising these data, the *minimum* point prevalence of FND may lie between 80 and 140/100 000, while the overall estimate based on all data sources varies more widely between 50 and 1600/100 000. To put this in context, based on the Global Burden of Disease Study, the prevalence worldwide of multiple sclerosis is 30/100 000, of motor neuron disease is 5/100 000 and of brain and other CNS cancers is 11/100 000,^65^ while the prevalence of depression is 3440/100 000 and of anxiety is 3780/100 000.^66^

Based on the lower, more conservative estimate of prevalence determined above, we can use whole population data to estimate that within the UK (population 67.6 million in 2022)^67^, there are likely a *minimum* of 50 000–100 000 people living with FND, and possibly up to 1 million if higher prevalence estimates are used. Within the USA (population 334.9 million in 2023)^68^, at a *minimum* there are likely 265 000–480 000 people living with FND, and possibly up to 5 million people.

### Additional groups

Incidence and prevalence of FND may vary when considering special populations. A study by Garrett *et al* estimated the incidence and prevalence of FND in active-duty US military personnel. They found 7644 incident cases over the years 2000–2018, with an estimated yearly incidence between 29.5/100 000 and 37.2/100 000,^69^ roughly two to three times that estimated in our review. This may reflect the lower age group or military setting but was a particularly interesting study that did not meet criteria for this review. Another scoping review examining the frequency of functional seizures in forcibly displaced persons noted one study showing an incredibly high FND symptom burden, with 28% of women experiencing functional seizures.^70^ While direct comparison of these data with other incidence and prevalence studies included in this review is difficult due to methodological differences, it demonstrates that the rate of FND in vulnerable populations may potentially be significantly higher than rates reported in the general population.

### Strengths and limitations

One major strength of this study was its inclusiveness of studies considered, particularly in reporting on data from studies looking at different FND symptom subtypes, which are often siloed in the literature (eg, movement disorder vs functional seizures). We additionally used studies of FND frequency, from which we extrapolated incidence and prevalence estimates, thus incorporating valuable data that otherwise get ignored. Studies from a wide range of geographical regions and both high-income and low-income countries were included, which also reflects a wide variety of cultures. Limitations of this study include the reliance on indirect methods of calculating incidence and prevalence rates for FND, due to the challenges posed by the source data. Data extrapolation, using functional seizures to calculate overall prevalence of FND, also assumed that 30% of patients with FND present with functional seizures. While this was based on a number of studies and seemed a reasonable estimate, a systematic search was not conducted to thoroughly verify the accuracy of this percentage, nor were studies evaluated for quality or bias. Most studies did not include data on patients with functional dizziness (or persistent postural-perceptual dizziness, the most common type of chronic dizziness^71^) or functional cognitive disorder, two newly recognised FND subtypes,^72^ which likely led to an underestimate of FND cases. Furthermore, functional speech and communication disorders and functional movement disorder subtypes were not included specifically in the search terms. While a number of studies did include patients with these symptoms, and we are unaware of any recent reviews focusing solely on these specific symptom subtypes, it is possible that some data were missed. However, we believe it is unlikely that this would have altered the overall results. With respect to generalisability of results, it is difficult to determine due to the heterogeneity of the data. Incidence studies were only available from high-income countries, which may not be generalisable to middle- and low-income countries. For the purposes of this review, only one database (PubMed) was searched. While in the authors’ experience additional database searches are unlikely to yield further articles on the topic of FND, it is possible that some articles were not included in this study due to the failure to search other databases.

### Future directions

Future studies aimed at estimating FND incidence or prevalence should ideally use the current DSM-5 diagnostic criteria and include all motor FND (including both weakness and movement disorders) cases as well as cases of functional seizures. Consideration should also be given to including newer FND subtypes, such as PPPD and functional cognitive disorder. Delineating these cases from patients with primary chronic pain disorders, fatigue and functional somatosensory symptoms alone (for which there are no robust rule-in signs) would capture a large proportion of cases while minimising the likelihood of including patients who may fit better into another diagnostic category (eg, fibromyalgia). Including multiple sources of data (eg, emergency care, neurology, psychiatry) would help capture the majority of cases. Consideration could also be given to including data from neuro-ophthalmology clinics and neurotology clinics in order to capture cases of PPPD and functional visual disorders. Cases are best ascertained by a neurologist or neuropsychiatrist with training in identifying this disorder.

## Data Availability

All data relevant to the study are included in the article or uploaded as supplementary information.
